# Managing robotics in the bioanalytical/metabolic environment of a pharmaceutical company

**DOI:** 10.1155/S1463924691000044

**Published:** 1991

**Authors:** Stanley Kushinsky

**Affiliations:** Syntex Research Palo Alto California 94304 USA


Journal of Automatic Chemistry, Vol. 13, No. 1 (January–February 1991), pp. 13–16

### Managing robotics in the bioanalytical/metabolic environment of a pharmaceutical company*

**Stanley Kushinsky**
*Syntex Research, Palo Alto, California 94304, USA*

### Introduction

About 5 or 6 years ago the author became interested in the possibility of using robotics to facilitate the performance of some routine analytical procedures. Based on a number of published articles, as well as on descriptive literature provided by the two primary manufacturers of relevant robotic equipment, it appeared that at least some of the procedures being performed manually could be done more efficiently by means of a robotic system. Not long thereafter, at demonstrations of two different robotic systems, the robotic hand dropped a test-tube—despite a diminished enthusiasm for robotics following these two incidents, the author asked a member of his staff to find out whether or not a robotic system, when it was not dropping test-tubes, could be of use to Syntex Research. The evaluations, conducted in collaboration with representatives from the manufacturers, were based on hypothetical comparisons of manual and robotic performance of two different types of procedures. One was an RIA and the other was an HPLC procedure that included a complex extraction sequence. The conclusions reached on completion of those '*Gedanken*' exercises was that performance of the RIA procedure robotically would not be cost effective and that the extraction procedure could not be accommodated by either of the robotic systems. Within the next couple of years, the Zymark Corporation made several important improvements in robotic technology. Among these were: (1) an increase in the rate of movement of the robotic arm; (2) availability of a greater variety of robotic operations; (3) substantial simplification of the required programming; (4) improved overall reliability; and (5) better control of the tactile strength of the robotic hand. In view of these improvements, the staff at Syntex Research reinvestigated the possible advantages of using robotics for performance of two procedures consisting of the steps outlined in table 1.

On completion of the evaluation, which included a rough estimate of payback period (to be defined shortly), it was apparent that the use of robotics was a viable alternative. This conclusion, perhaps garnished with some wishful thinking, led to the initiation of a significant effort directed at selecting the most suitable robotic system for performance of the targeted procedures and for obtaining the required funding. Selection of an appropriate system was relatively straightforward, since most of the required components had been specified in the course of the preliminary evaluation. Preparation of a justification for capital spending was more arduous. Among the directly pertinent and peripheral issues to be addressed were:

*Table 1. Procedures investigated using robotics.*

| HPLC procedure | HPLC/RIA procedure |
|---|---|
| Dispense plasma | Dispense plasma |
| Add internal standard | Add internal standards |
| Add buffer | Vortex mix |
| Vortex mix | Add extracting solvent |
| Condition column | Tumble mix |
| Load plasma | Centrifuge |
| Wash column | Transfer and evaporate organic phase |
| Elute analyte(s) | Dissolve residue/vortex mix |
| Evaporate solvent | Evaporate solvent |
| Dissolve residue | Redissolve in small volume |
| Perform on-line HPLC (signal output to data system) | Perform on-line HPLC (separation of analytes for subsequent RIA) |

(1) How much will the robotic system cost?

(2) What is the projected payback period?

(3) Is a suitable location available for it?

(4) What are the projected costs for any modifications to facilities that may be required?

(5) Will the robotic system lead to a reduction in staff size?

(6) What effect will the use of robotics have on the character of the work force?

(7) What criteria should be considered in selection of personnel for the robotics project?

(8) What caveats need to be taken into account?

(9) What future directions are anticipated for robotics in our facilities?

Summaries of the responses prepared to address these issues, in some cases with additional commentary, follow in sequence.

### Cost

The initial estimate of the cost of the desired system that was used for budgeting purposes was approximately \$80 000. However, as a result of several very significant

* Abstract published in *Journal of Automatic Chemistry*, Vol. 12, No. 6.

improvements to the basic system that were made by the manufacturer after the initial quotation had been obtained, the cost of the system considered to be most suited to meet Syntex's requirements and the one that ultimately was ordered, had increased to approximately $100 000. This difference in cost had to be made up by using funds that had been allocated previously to other capital items. Including the cost of an HPLC system with fraction collector that is used in conjuction with the robotic system, and a few incidental items such as a backup power supply/orderly shutdown device and a weighing station that were purchased subsequently, the total cost of the robotic station was close to $130 000, plus about $10 000 for some very useful, 'one time only', special programming by Zymark.

### Payback period

One of the most difficult variables to estimate accurately for robotic systems is the payback period, opinions to the contrary notwithstanding. The payback period is defined as the time required to recover the cost of the equipment through improved productivity. Among the factors contributing to the difficulty of assessing the payback period are the following.

Estimating the analytical throughput for a robotic procedure that has not yet been reduced to practice generally involves some questionable assumptions. Even the estimates of throughputs for routine non-robotic procedures are subject to considerable uncertainty as a result of work interruptions and episodic procedural failures. Both types of estimates tend to be idealized and the deviations from the idea can have a significant effect on the actual throughputs obtained after prolonged periods of observation for robotic and non-robotic procedures. Despite these reservations, a payback period of approximately 2 years was estimated, assuming that the saving in salary and benefits for one chemist during this period would be comparable to the cost of the basic robotic system, without the HPLC. Subsequent, more meaningful estimates of payback period done after 1–2 years of use of the robotic system, during which many performance tests were conducted and numerous modifications made to incorporate additional features, led to the values of 2·2 to 3·3 years, as shown in table 2. Robotic throughput refers to overall throughput for the chemist plus the robot. The equation used for these calculations ignores factors such as robotic set-up time, downtime, overhead expenses and tax consequences, which collectively could well balance one another to have a negligible impact on the effective payback period.

### Location

None of the existing laboratories contained an area suitable for installation of the proposed robotic system without costly modifications to the facilities. The alternative selected was to locate the robotic system in an office adjacent to one of the laboratories. This solution involved finding an office for the displaced individual and installation of some additional electrical circuits, as well as some flexible ducting to an existing fume-hood system for venting of potentially hazardous/biohazardous materials, but no costly modifications. In addition to this being the least costly alternative, it provided an important advantage in that there was no need to justify costly modifications to facilities based only on theoretical considerations, promises and imaginative brochures. After the first robotic systems has proven to be of value, requests for funds to perform major modifications to facilities for installation of additional robotic systems are much easier to justify.

### Staff size and character

If robotic technology is set up to operate in an efficient manner, and the workload remains relatively constant, the size of the required work force should decrease. The magnitude of the decrease in staff size depends on such factors as the relative efficiency of the manual and the robotic methods and how productively the operators' time is spent while robotic procedures are being performed. Also quite likely to change is the character of the work force – from one more comfortable with near total control over all operations to one that is more goal oriented, with little or no concern as to how the goals are achieved. When the idea of introducing robotics into the workplace is first broached, some individuals will feel threatened. They may express a negative attitude about robotics, before having any substantive basis for drawing conclusions concerning the potential usefulness of this

*Table 2. Payback period.*

| Procedure | Projected annual sample throughout per chemist Robotic | Non-robotic | Payback period* (years) |
|---|---|---|---|
| HPLC[a] | 20·8 K | 10·4 K | 2·2 |
| HPLC/RIA[b] | 6·2 K[c] | 3·6 K | 3·3 |
| | | | Average 2·75 |

$$\text{* Payback period} = \frac{\text{Cost of robotic system}}{\left[\dfrac{\text{Robotic throughput}}{\text{Non-robotic throughput}} - 1\right] \times [\$50\text{ K annual salary per chemist}]}$$

Cost of robotic system: [a] $112 000; [b] $120 000. [c] Zymate system is used 3 days/week.

technology. Others may express exhilaration at the prospect of being relieved of much of the drudgery involved in the performance of routine procedures, tempered perhaps by some concern over job security. It is absolutely essential to provide adequate background information to all personnel who may be affected by a decision to implement robotics on more than a trial basis. With proper attention to education, those individuals who initially feel most threatened by the prospect of robotics could easily become the most enthusiastic proponents of robotics. Conversely, those who initially appear to be most eager to use robotics may not ultimately be very comfortable with or successful in a robotic environment. A chronic problem in the (bio)-analytical laboratory has been that a substantial number of chemists after many years of working at the bench become frustrated at the prospect of having to spend the remainder of their career performing procedures that consist of a series of relatively simple steps. The satisfaction that comes from seeing the end result of their efforts provides adequate incentive for some, but not for all, to continue their careers at the bench. Those who become frustrated with their jobs generally manage to find different careers, thereby opening up positions for others to fill. Increased use of robotics may provide sufficient incentive for many individuals to continue their careers in (bio)analytical chemistry. It also seems reasonable to expect that students contemplating possible career choices might be more inclined to select (bio)-analytical chemistry if they were not faced with the prospect of having to perform a seemingly endless series of very routine and tedious operations each day at work. This choice is particularly critical for individuals who are aware of the generally limited prospects for career advancement if they terminate their formal education short of a doctorate. Through the use of robotics in the (bio)analytical laboratory, an entirely new area of opportunity becomes available for individuals who would find attractive the possibility of using their training in both chemistry and computer science for a rewarding career in which most of what they consider to be 'mindless' tasks would be done by a mechanical robot under their control.

### Selection of personnel

Undoubtedly, the most critical factor in the establishment of a successful robotic facility is assigning the task to an individual who possesses the appropriate blend of talents and ensuring that all needed support is provided. Among the desired characteristics are: extensive experience in the development and performance of analytical procedures; practical gadgeteer; substantial familiarity with computers/programming; innovativeness; resourcefulness and enthusiasm. In addition, having a good rapport with key personnel in the machine and maintenance shops can be extremely beneficial, if not indispensable. (At Syntex Reseach, all of the individuals in these shops have been more than willing to provide their support, particularly after they have seen the robot in operation.) If a suitable individual, who possesses all or most of these attributes, is not available within the organization, the only viable alternatives are to recruit someone from elsewhere, or to abandon the idea of using robotics. Assignment of the task of implementing a robotic system as a side project to someone who possesses all of the desired characteristics will not suffice. Robotics must be viewed as a full-time undertaking, once the decision to proceed has been made. It also is very desirable to have the individual who will be responsible for implementation of the system to participate in the initial evaluation of alternatives and in the planning of the system. After the first system is fully operational, it certainly is not necessary for the personnel who will use the equipment routinely to possess all of the aforementioned characteristics. It was recently found that an individual who had absolutely no prior experience with robotics was able to learn to use the robotic system after only about 2 days of training. In part, this was possible because the system was set up with a menu that allowed the operator to select the desired assay, load the specimens, reagents and supplies into the unit and press the 'run' button. Credit for this accomplishment clearly must go to the person who configures the system in this most efficient and 'user-friendly' manner.

### Caveats

When preparing a justification for the first robotic system that is to be installed within a particular facility, there is a great temptation to overstate the potential benefits that may be derived through its use. In such cases, if any significant delays are encountered in implementing the system, and upper management begins to track these delays with possible undue concern, the entire operation could be jeopardized. Alternately, if the potential benefits of robotics are understated, the activity may never be approved. The most sensible approach is to make the best effort to be as realistic as possible in describing the potential benefits of robotics, keeping in mind the fact that there are some automated operations that can be performed more efficiently by means of specialized equipment than by a general robotic system such as that described here. One such operation is pipetting for EIAs or RIAs. Undoubtedly, there are many others. Also worthy of mention in this regard is the temptation to use robotics for all portions of a procedure, without considering the possibility that one or more steps could be done more efficiently with some manual intervention. One example of such a situation was encountered recently during the evaluation of a Benchmate system for a procedure involving a complex solid-phase extraction step. In this case, it appears that it will be more efficient to use the Benchmate through the extraction step and then to use a Turbovap unit to remove the solvent before proceeding to the HPLC step, and, finally, to use the Benchmate to dissolve the residues and to inject the samples. Although no final decision has been made, it is likely that for this, and other projects, some steps will be performed manually. In other instances, such as when the total number of specimens to be assayed on a particular day is relatively small and the experimental design precludes waiting for additional samples to be accumulated, the use of robotics may not be justified. An appropriate strategy might be to use a system such as the Zymate only when a Benchmate (or equivalent) will not suffice and to use neither for steps that can be done better manually.

### Future directions

One question that arose early in the author's thinking about robotics is whether it would be better to have a centralized robotic unit or to spread the robotic operations throughout the facilities. A recently ordered second Zymate and a Benchmate will be located in the laboratory adjacent to the existing unit and the current operators will provide training and support to various other users of the equipment as needed. As future systems are added and more chemists become familiar with the operation and maintenance of these systems, the units will be placed at appropriate locations throughout the facilities at Syntex. Nevertheless, there is the possibility that some activities may for the foreseeable future remain within the 'robotic methods development' group and be transferred elsewhere once the methods are established. If methods are set up by specifying a range of conditions for each step from which users can select the one that is most suited to a particular analyte, a single generic method would be suitable for many different analytes, thereby providing enormous flexibility. This tactic would enable a more widespread use of robotics by non-experts and simplify the development of methods for new analytes. Thus far, most attention has been devoted to relatively minor modifications of manual methods to make them applicable to robotic technology. One extremely important future use for robotic systems at Syntex involves the development of new methods, or the optimization of methods through the evaluation of different analytical conditions robotically.

### Closing comments

Based on our experiences with robotics during the past couple of years, it is apparent that most, if not all, manual procedures can be performed more efficiently and cost-effectively through the use of robotics. Thus far, the percentage of operations at Syntex that have become robotic is relatively small. Within the next few years, it is very likely that nearly all routine procedures will be developed and performed with extensive use of robotic technology. The change to a robotic environment is inevitable, given the rapidly escalating cost of labour and health benefits, the desire by management to improve productivity, and the ready availability of all required technology. The next generation of (bio)analytical chemists will find it difficult to imagine how their predecessors managed to survive the monotony of a non-robotic environment.

